# Baicalin Protects Mice Brain From Apoptosis in Traumatic Brain Injury Model Through Activation of Autophagy

**DOI:** 10.3389/fnins.2018.01006

**Published:** 2019-01-09

**Authors:** Jiang Fang, Yihao Zhu, Handong Wang, Bailu Cao, Maoxing Fei, Wenhao Niu, Yuan Zhou, Xiaoliang Wang, Xiang Li, Mengliang Zhou

**Affiliations:** ^1^Department of Neurosurgery, Jinling Hospital, Nanjing, China; ^2^School of Medicine, Southeast University, Nanjing, China; ^3^School of Medicine, Nanjing University, Nanjing, China; ^4^Jinling Clinical Medical College, Nanjing Medical University, Nanjing, China; ^5^Department of Endocrinology, Jinling Hospital, Nanjing, China; ^6^Department of Neurosurgery, Jiangsu Provincial Second Chinese Medicine Hospital, Nanjing, China

**Keywords:** Baicalin, traumatic brain injury, autophagy, apoptosis, mitochondrial apoptosis

## Abstract

Autophagy is associated with secondary injury following traumatic brain injury (TBI) and is expected to be a therapeutic target. Baicalin, a neuroprotective agent, has been proven to exert multi-functional bioactive effects in brain injury diseases. However, it is unknown if Baicalin influences autophagy after TBI. In the present study, we aimed to explore the effects that Baicalin had on TBI in a mice model, focusing on autophagy as a potential mechanism. We found that Baicalin administration significantly improved motor function, reduced cerebral edema, and alleviated disruption of the blood-brain barrier (BBB) after TBI in mice. Besides, TBI-induced apoptosis was reversed by Baicalin evidenced by Nissl staining, terminal deoxynucleotidyl transferase dUTP nick end labeling (TUNEL) assay, and the level of cleaved caspase-3. More importantly, Baicalin enhanced autophagy by detecting the autophagy markers (LC3, Beclin 1, and p62) using western blot and LC3 immunofluorescence staining, ameliorating mitochondrial apoptotic pathway evidenced by restoration of the TBI-induced translocation of Bax and cytochrome C. However, simultaneous treatment with 3-MA inhibited Baicalin-induced autophagy and abolished its protective effects on mitochondrial apoptotic pathway. In conclusion, we demonstrated that Baicalin enhanced autophagy, ameliorated mitochondrial apoptosis and protected mice brain in TBI mice model.

## Introduction

Traumatic brain injury (TBI) is a modern day public health problem known for its high morbidity, mortality, disability and huge medical costs (Brooks et al., 2013). The pathophysiology of TBI involves primary brain insult and secondary injury. The primary injury occurs at the moment of impact and is irreversible. The secondary damage, including calcium overload, oxidative stress, inflammation, and apoptosis, exacerbates neuronal loss and is reversible (Cornelius et al., 2013; Mustafa and Alshboul, 2013). Despite the research regarding the treatment of TBI, there is no effective treatment to alleviate the secondary insult thereby improving the outcome of TBI patients. Macroautophagy (hereafter called autophagy) is a highly conservative intracellular process that is essential for eliminating abnormal proteins or organelles to maintain intracellular homeostasis under stress situations (Mizushima et al., 2008). This cellular degradation process is implicated in cell apoptosis in many central nervous system (CNS) diseases, including TBI (Lipinski et al., 2015; Cortes and La Spada, 2018; Fowler and Moussa, 2018). Signs of autophagy activation are observed among post-traumatic secondary pathological responses and several neuroprotective drugs are proven to attenuate TBI-induced secondary neuronal death via enhancement of autophagy (Xu et al., 2014; Zhang et al., 2016; Chen et al., 2018b). Although, elimination of damaged mitochondria via autophagy may be a potential mechanism (Ding et al., 2015), the precise mechanism remains unclear.

Baicalin (7-D-glucuronic acid-5,6-dihydroxyflavone) is a major flavonoid in traditional Chinese medicinal herb that is isolated from the radix of Scutellaria baicalensis (Ma et al., 2009). It freely crosses the blood-brain barrier (BBB) (Tarrago et al., 2008) and exerts multiple neuroprotective effects, including anti-inflammation (Shi et al., 2017), anti-apoptosis (Zuo et al., 2016), and anti-oxidation (Hong et al., 2017). Besides, the relationship between Baicalin and autophagy is previously documented. It has been reported that Baicalin could induce macroautophagy in human hepatocellular carcinoma SMMC-7721 cells, human bladder cancer T24 cells, and macrophages infected with mycobacterium tuberculosis, which exerts anti-cancer and anti-inflammation properties (Zhang et al., 2012, 2017; Lin et al., 2013). However, whether Baicalin activates autophagy in brain after TBI has not been studied. The present study was carried out to investigate whether Baicalin activates autophagy and protects brain against apoptosis in TBI mice model.

## Materials and Methods

### Animals

Male Institute of Cancer Research (ICR) mice (Animal Experimental Centre of Nanjing Medical University, Jiangsu, China), weighing 28–32 g, were housed under controlled environmental conditions with a 12 h light/dark cycle, with free access to food and water and were acclimated for at least 4 days before experiments. All experimental protocols were in line with the Guide for Care and Use of Laboratory Animals by the National Institutes of Health (NIH) and were approved by the Animal Care and Use Committee of Southeast University, Nanjing, China.

### TBI Model

The TBI model in our study was based on the weight-drop model described by Flierl et al. (2009). Briefly, the mice were anesthetized (10% chloral hydrate, 4 ml/Kg, i.p.) and then placed onto the platform under the weight-drop device. A 1.5 cm midline longitudinal scalp incision was made to expose the skull. After the impact area (1.5 mm lateral to the midline on the mid-coronal plane) was confirmed, a 200 g weight was released from a height of 2.5 cm. The scalp wound was closed and the mice were returned to quondam cages. The mice in sham group underwent the same procedure except the weight drop.

### Experimental Design

**FIGURE 1:**
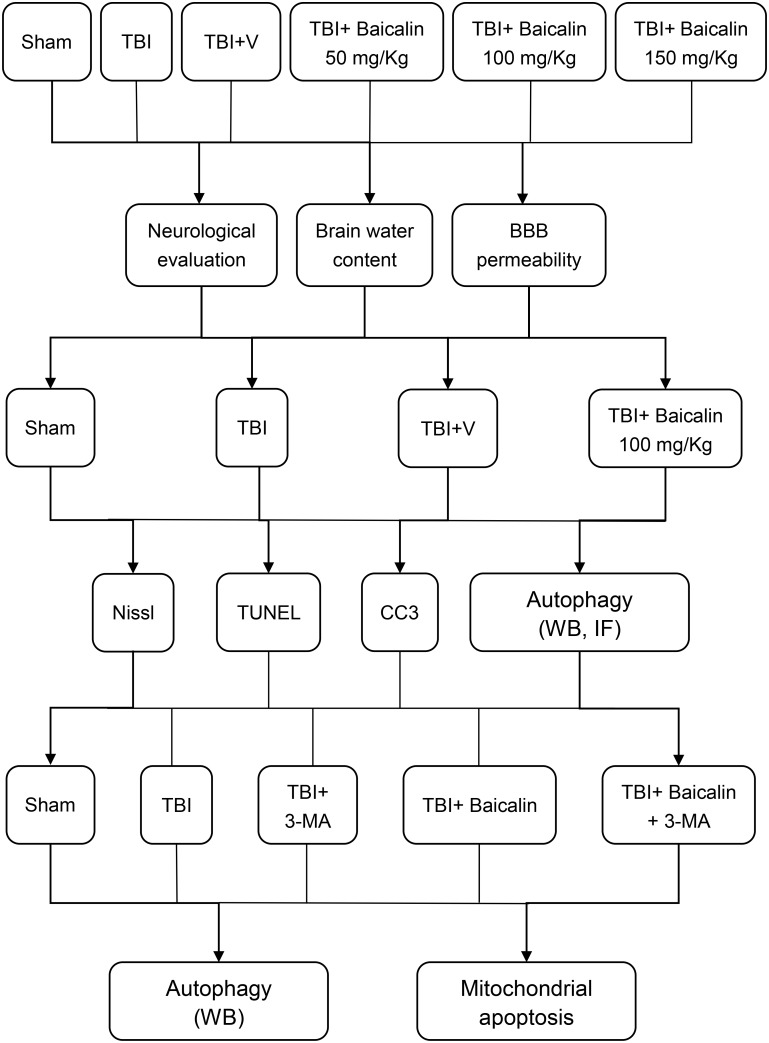
The diagram demonstrating the experimental design throughout the study.

To explore Baicalin-mediated neuroprotective effects and activation of autophagy after TBI, we randomly divided mice into four groups: sham, TBI, TBI+ vehicle, TBI+ Baicalin (Figure 1). The mice in TBI+ Baicalin group were administrated with Baicalin (Sigma-Aldrich, St. Louis, MO, United States) intraperitoneally 30 min after onset of TBI. The dosage was chosen from previous papers on TBI and cerebral ischemia models (Tu et al., 2009; Cao et al., 2011; Fang et al., 2018). The mice in the TBI+ vehicle group were administered isotonic saline. For neurologic evaluation, the mice were injected with Baicalin or isotonic saline 30 min and every 24 h after TBI.

To further confirm the protective role of autophagy induced by Baicalin, a commonly used autophagy inhibitor, 3-MA (Sigma-Aldrich, St. Louis, MO, United States) was utilized. Animals were randomly assigned to five groups: Sham, TBI+ vehicle, TBI+ 3-MA, TBI+ Baicalin, TBI+ Baicalin+ 3-MA. The mice in 3-MA group or the vehicle group were injected 2 μl solution of 3-MA (400 nmol dissolved in 2 μl vehicle) or the vehicle (1 μl DMSO dissolved in 1 μl saline) by intracerebroventricular injection to the left lateral ventricle (bregma; 1.0 mm lateral, 0.3 mm posterior, 2.6 mm deep) 30 min prior to TBI according to previous research (Wang et al., 2012). All mice from each group were euthanized 24 h post-TBI except those for neurologic evaluation. Mice were deeply anesthetized via inhalation of isoflurane at 24 h after TBI and perfused transcardially with 0.9% normal saline solution. The brain was harvested for subsequent biochemical and histological analyses.

### Brain Water Content and Blood-Brain Barrier (BBB) Permeability

The wet-dry method was utilized to evaluate brain edema according to a previous study (Zhang et al., 2016). The brains were obtained from deeply anesthetized mice 24 h post-TBI. Then, the brain stem and cerebellum were removed. The ipsilateral brain tissue was weighed immediately to acquire the wet weight (WW), and then dried for 3 days at 76°C to obtain the dry weight (DW). The formula was as follows: (WW-DW)/WW × 100%.

BBB disruption was assessed by quantifying Evans blue dye extravasation (Chen et al., 2018a). Briefly, 24 h after TBI, 2% Evans blue dye (4 ml/Kg) was injected intravenously to mice and allowed to circulate for 4 h prior to sacrifice. Then, the brain was perfused by PBS, weighed, and homogenized in a solution containing 1 ml 50% trichloroacetic acid. Evans blue absorbance of the supernatant followed by centrifuge was determined at 630 nm. The extravasation of Evans blue dye was calculated as micrograms per gram brain tissue based on the standard curve.

### Neurobehavioral Evaluation

The neurological level of injury was assessed at 1, 3, and 7 days after TBI by neurological severity scoring (NSS) and grip tests. The NSS method consists of 10 different tasks including the motor, balance, and reflex test scores (Flierl et al., 2009). One point is given for the inability to perform each task. Therefore, a higher score represents a more severe brain injury (Table 1). The grip test score was quantified by a five-point scale (Bermpohl et al., 2006). In short, mice were placed onto a metal wire (45 cm long) that was suspended 45 cm above a foam pad and were allowed to traverse the wire for 1 min. The evaluation criterion is showed in Table 2. The grip test was conducted in triplicate, and a total value was calculated for each mouse. All neurobehavioral tests were performed by an investigator who was blind to the experimental groups.

**Table 1:** Neurologic severity score (NSS).

Task	Description	Point (success/failure)
Exit circle	Ability and initiative to exit a circle of 30 cm diameter within 3 min	0/1
Monoparesis/hemiparesis	Paresis of upper and/or lower limb of the contralateral side	0/1
Straight walk Alertness	Initiative and motor ability to walk straight	0/1
Startle reflex Innate reflex	The mouse will bounce in response to a loud hand clap	0/1
Seeking behavior	Physiological behavior as a sign of “interest” in the environment	0/1
Beam balancing	Ability to balance on a beam of 7 mm width for at least 10 s	0/1
Round stick balancing	Ability to balance on a round stick of 5 mm diameter for at least 10 s	0/1
Beam walk: 3 cm	Ability to cross a 30 cm long beam of 3 cm width	0/1
Beam walk: 2 cm	Same task, increased difficulty on a 2 cm wide beam	0/1
Beam walk: 1 cm	Same task, increased difficulty on a 1 cm wide beam	0/1
Maximal score 10		


**Table 2:** Grip test scoring.

Description	Points
Unable to remain on the wire for less than 30 s	0
Failed to hold on to the wire with both sets of fore paws and hind paws together	1
Held on to the wire with both forepaws and hind paws but not the tail	2
Used its tail along with both forepaws and both hind paws	3
Moved along the wire on all four paws plus tail	4
Scored four points also ambulated down one of the posts used to support the wire	5


### Nissl Staining and TUNEL Analysis

For histological examination, mice were killed 24 h after TBI. Damaged neurons were detected by the method of Nissl staining as previously described (Ding et al., 2017). Normal neurons showed regular cell morphology and round nuclei. Damaged cell featured with irregular neuronal cell body, shrinking and hyperchromatic nucleus, and dried-up cytoplasm with vacuoles (Liang et al., 2018).

Apoptotic cells were determined with a Roche TUNEL detection kit (Roche, Indianapolis, IN, United States) according to the manufacture’s instruction. Briefly, coronal sections (6 mm) were deparaffinized, rehydrated, washed with PBS followed by digesting for 15 min in proteinase K, then incubated with labeling solution containing TUNEL reaction fluid for 50 min at 37°C. Streptavidin peroxidase (HRP) diluted to 1:40 was applied to detect the labeled nucleotides. The sections were stained with DAB as chromogen. The apoptotic cell had a shrinkage cell body and a condensed nucleus that was stained brown. Cell counting was conducted by two investigators blind to group assignment. Six random fields (under 400× magnification) surrounding the injury site were chosen from each coronal section. Four sections of each mouse were used to calculate the final average percentage, which was regarded as the numerical value of each sample.

### Western Blot Analysis

The tissue located over the injure site (both contusion and penumbra) was collected 24 h after TBI. Total protein extraction was acquired using the Total Protein Extraction Kit (Beyotime Biotech, Nantong, China) according to the manufacture’s instruction. Mitochondrial and cytosolic proteins were isolated with the Tissue Mitochondria Isolation Kit (Beyotime Biotech, Nantong, China) according to the kit instruction (Ding et al., 2015). Nuclear and cytoplasmic proteins were isolated via the Nuclear and Cytoplasmic Protein Extraction Kit (Beyotime Biotechnology, Shanghai, China) according to manufacturer’s instructions (Fang et al., 2018). Protein concentrations were determined via the BCA method. Equal amounts of protein were subjected to 10–12% sodium dodecyl sulfate-polyacrylamide gel electrophoresis (SDS-PAGE) followed by being transferred to PVDF membranes (Millipore, Bedford, MA, United States). The membranes were blocked with 5% freshly prepared skim milk-TBST for 2 h and then incubated overnight at 4°C with following primary antibodies: cleaved caspase-3 (1:1000, Cell Signaling Technology, Beverly, MA, United States), LC3 (1:1000 Novus Biologicals, Minneapolis, MN, United States), Beclin 1 (1:1000 Novus Biologicals, Minneapolis, MN, United States), p62/SQSTM1 (1:1000 Abcam, Cambridge, United Kingdom), Bax (1:200, Santa Cruz Biotechnology, Santa Cruz, CA, United States), cytochrome C (1:1000 Abcam, Cambridge, United Kingdom), COX IV (1:1000, Cell Signaling Technology, Beverly, MA, United States), β-actin (1:5000, Bioworld Technology, St. Louis, MO, United States), AMPK (1:200, Proteintech Group, Chicago, United States), p-AMPK (1:1000, Cell Signaling Technology, Beverly, MA, United States), mTOR (1:1000, Cell Signaling Technology, Beverly, MA, United States), p-mTOR (1:1000, Cell Signaling Technology, Beverly, MA, United States), p53 (1:500, Proteintech Group, Chicago, IL, United States), and H3 (1:1000, Cell Signaling Technology, Beverly, MA, United States). Subsequently, the membranes were incubated with the corresponding horseradish peroxidase-linked secondary antibodies for 1 h. The signal was visualized using ECL substrate (Millipore, Billerica, MA, United States). Band density was quantified using Image J Software.

### Immunofluorescence

Immunofluorescence double staining of LC3 and NeuN was conducted to detect the level of autophagy. About 24 h after TBI, brain samples were obtained, fixed in 4% formalin overnight, then cut into 6 μm sections. After treated with blocking buffer (10% normal goat serum in PBS containing 0.01% Triton X-100) for 1 h, the sections were incubated with LC3 (1:100 Novus Biologicals, Minneapolis, MN, United States) and NeuN (1:100; Millipore, Bedford, MA, United States) overnight at 4°C. After being washed in PBST, the sections were incubated with appropriate secondary antibodies (1:200, Alexa Fluor 594) for 2 h. Then, washed by PBST, the sections were counterstained with DAPI for 4 min, rinsed with PBST, and cover slipped. Fluorescence was imaged on a Zeiss HB050 inverted microscope system.

We classified the cells with signal of LC3 puncta (caused by LC3 aggregation) around nucleus as LC3-positive cells or the cells with only diffuse LC3 signal as LC3-negative cells according to the paper by Viscomi et al. (2012). The LC3-positive ratio was the number of LC3-positive neurons, normalized to the total number of neurons. Six fields (under a microscope 400×) around the injury site in each coronal section were selected. Four sections of each mouse were used to calculate the final average percentage, which was regarded as the numerical value of each sample.

### Statistical Analysis

All data were expressed as mean ± SD. Statistical analysis among groups was performed by the one-way ANOVA followed by Tukey’s *post hoc* tests except for neurological function score, which was analyzed by two-way ANOVA with repeated measures followed by Bonferroni post-tests. The SPSS 19.0 software (SPSS Inc., NY, United States) was used for the statistical analysis. *P* < 0.05 was considered statistically significant.

## Results

### Baicalin Provided Neuroprotection After TBI

**FIGURE 2:**
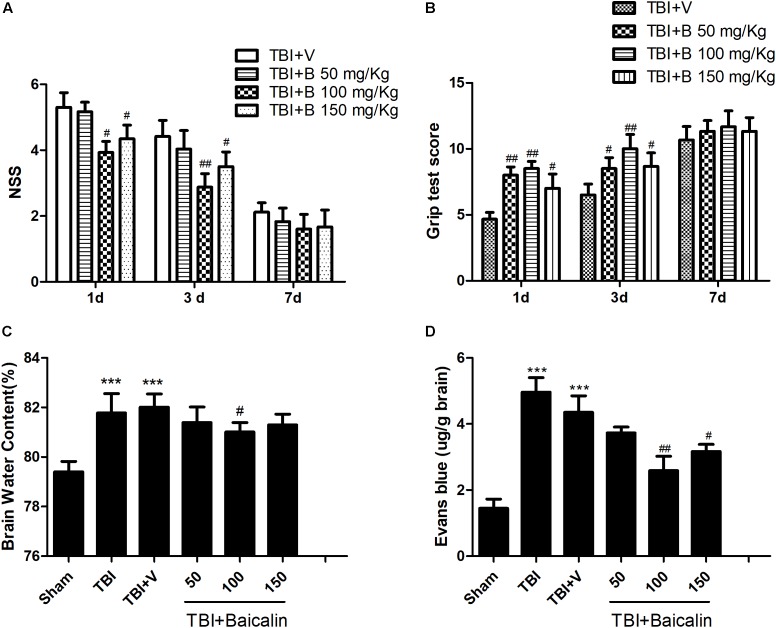
Baicalin protected mice brain after TBI. **(A,B)** Compared with vehicle group, Baicalin administration significantly improved mice motor function at 1 and 3 days after TBI. A dose of 100 mg/kg exerted the best effects. **(C,D)** Compared with TBI+ V group, TBI induced increase of brain water content and extravasation of Evans blue dye were alleviated by administration of Baicalin. Dose of 100 mg/kg had the best effects. Data are represented as mean ± SD (*n* = 6 per group). ^∗∗∗^*P* < 0.001 compared with sham group; ^#^*P* < 0.05 and ^##^*P* < 0.01 compared with the TBI+ V group.

To determine whether Baicalin provides neuroprotective effects in TBI mice, we used NSS and grip test score to assess mice motor function at 1, 3, and 7 days after TBI. The scores of sham animals were almost zero in NSS test and 15 scores in grip test at the three time points. Besides, there is no significant difference between TBI and TBI+ V groups. Thus, the data in sham and TBI groups are not showed. As shown in Figure 2, compared with vehicle group, the animals treated with Baicalin performed much better at 1 day. The difference was still detectable at 3 days, but diminished at 7 days (*p* > 0.05; Figures 2A,B).

To further confirm the protective effects, we evaluated mice brain water content and BBB integrity using the wet-dry weight method and the Evans blue method. Compared with sham group, the brain water content was significantly increased in TBI and TBI+ V group (*p* < 0.001 and *p* < 0.001; Figure 2C). However, the water content of the Baicalin-treated group was markedly lower than that of vehicle group (*p* < 0.05; Figure 2C). The results of BBB permeability indicated that TBI-induced extravasation of Evans blue dye was decreased after Baicalin administration (*p* < 0.01 and *p* < 0.05; Figure 2D), demonstrating that Baicalin alleviated the BBB disruption after TBI. These tests indicated that the dosage of 100 mg/Kg Baicalin had the best therapeutic effects, which was used in the subsequent experiments.

### Baicalin Reduced Neuronal Apoptosis Following TBI

**FIGURE 3:**
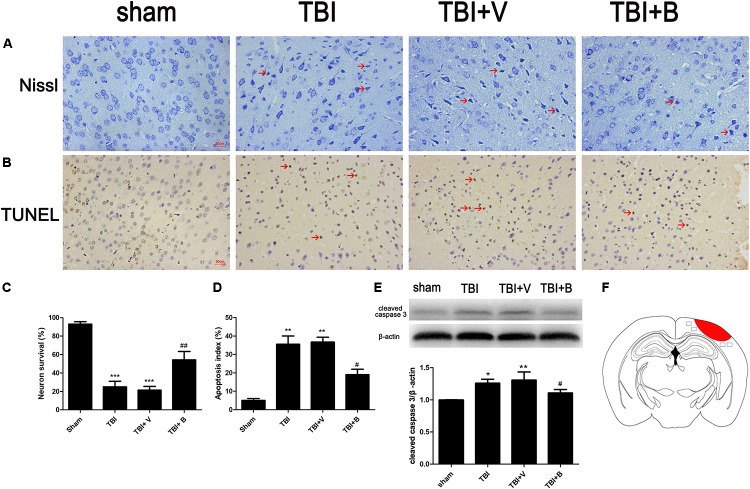
Baicalin suppressed neural apoptosis induced by TBI. **(A,C)** Representative Nissl staining and the percentage of neuron survival in four groups. The red arrow indicated damaged neurons. **(B,D)** Representative TUNEL staining and the percentage of apoptotic cells in four groups. The red arrow indicated apoptotic cells. **(E)** The western blot band and semiquantitative analysis of cleaved caspase-3. **(F)** A schematic of a brain section after TBI. The area in red refers to injury site and areas in blue refer to regions detected for morphological examination. Data are represented as mean ± SD (*n* = 6 per group). ^∗^*P* < 0.05, ^∗∗^*P* < 0.01, and ^∗∗∗^*P* < 0.001 compared with sham group; ^#^*P* < 0.05 and ^##^*P* < 0.01 compared with the TBI+ V group. Scale bar: 50 μm.

Nissl staining was used to detect the damaged neurons in the ipsilateral cortex surrounding the injury site (Figure 3A). The neurons in sham group were clear and intact. However, in TBI and TBI+ vehicle groups, the damaged neurons were significantly increased (*p* < 0.001 and *p* < 0.001; Figure 3C), which had irregular neuronal cell bodies, shrinking and hyperchromatic nuclei. However, after Baicalin was applied, a significant lower percentage of neuron loss was detected as compared to vehicle group (*p* < 0.01; Figure 3C).

TUNEL staining showed that TBI caused significant cell apoptosis demonstrated by the shrinkage in the cell bodies and the condensed nuclei stained brown (*p* < 0.01; Figures 3B,D). However, compared to TBI+ V group, the apoptotic index was significantly decreased after Baicalin administration (*p* < 0.05; Figure 3D). Western blot analysis further confirmed that Baicalin downregulated pro-apoptotic protein, cleaved caspase-3 in TBI+ B group as compared with TBI+ V group (*p* < 0.05; Figure 3E). The data revealed that Baicalin exerted neuroprotection against TBI-induced apoptosis in mice model.

### Baicalin Enhanced Autophagy in the Brain Cortex Surrounding Injury Site

To further explore the mechanism of neuroprotection by Baicalin, we examined the levels of the three autophagy-associated proteins (LC3, Beclin 1, and p62) which usually were detected via western blot as autophagosome markers (Zhang and Wang, 2018). Microtubule-associated protein light chain 3 (LC3) was primarily synthesized as the form of proLC3, which then formed LC3I after cleavage. The LC3I then bound to phosphatidylethanolamine (PE) and became LC3II, which subsequently localized to phagophore and contributed to the elongation of isolation membrane (Kabeya et al., 2000). Beclin1 played a critical role in the formation of autophagosome through interactions with Vps34. The p62, served as a cargo protein, promoted the ubiquitination of cytoplasmic organoids to autophagosome and was degraded by autolysosome (Klionsky et al., 2016).

The AMPK/mTOR pathway is a classical signaling pathway in autophagy. The activation of mTOR could inhibit the autophagy, and phosphorylation of AMPK inhibited the activation of mTOR, so the activation of AMPK promotes the level of autophagy (He and Klionsky, 2009).

**FIGURE 4:**
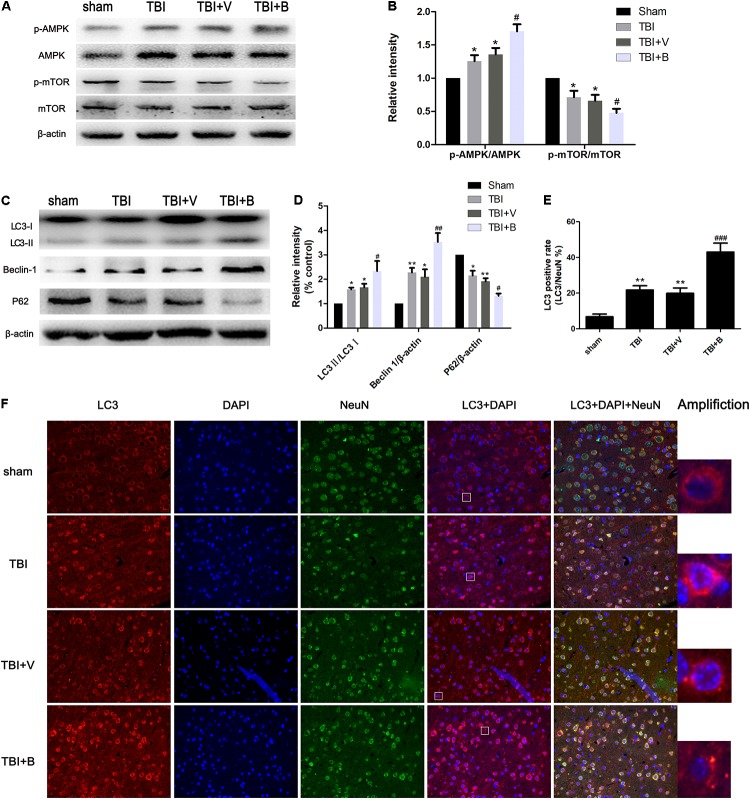
Baicalin enhanced autophagy following TBI. **(A,B)** The western blot band and semiquantitative analysis of p-AMPK, AMPK, p-mTOR, mTOR. **(C,D)** The western blot band and semiquantitative analysis of LC3, Beclin 1, and p62. **(E,F)** Representative immunofluorescence staining and the percentage of LC3 positive rate in four groups. LC3-II fluorescence signal diffused in sham group. However, in TBI and Baicalin-treated groups, more LC3-II aggregations were observed. White rectangles in respective group were amplified to show the typical cells with LC3-II aggregation. Data are represented as mean ± SD (*n* = 6 per group). ^∗^*P* < 0.05 and ^∗∗^*P* < 0.01 compared with sham group; ^#^*P* < 0.05, ^##^*P* < 0.01, and ^###^*P* < 0.001 compared with the TBI+ V group. Scale bar: 50 μm.

As shown in Figure 4, compared with sham group, the ratio of LC3 II/LC3 I as well the level of Beclin 1 were significantly increased in TBI (*p* < 0.05 and *p* < 0.01; Figure 4D) and TBI+ V (*p* < 0.05 and *p* < 0.05; Figure 4D) groups. Conversely, the protein level of p62 was dramatically decreased after TBI (*p* < 0.05 and *p* < 0.01; Figure 4D). The results indicated that TBI activated autophagy flux. More interestingly, 24 h after Baicalin injection, the changes were more obvious on LC3 II/LC3 I, Beclin 1, and p62 (*p* < 0.05, *p* < 0.01, and *p* < 0.05; Figure 4D). Besides, the protein expression of p-AMPK in the Baicalin group was significantly upregulated compared to the vehicle group, while the level of p-mTOR was downregulated between the two groups (Figures 4A,B). To sum up, the results demonstrated that Baicalin enhanced autophagy at least in part by activating the AMPK/mTOR pathway after TBI in the mice brain cortex.

Immunofluorescence staining exhibited consistent results with western blot (Figure 4F). TBI significantly increased the ratio of LC3 puncta (Amplification in Figure 4F) cells from approximately 7% to 21% as compared to sham group (*p* < 0.01; Figure 4E). Moreover, Baicalin injection further increased the LC3 positive rate to 43% in Baicalin group compared with vehicle group (*p* < 0.001; Figure 4E).

### 3-MA Reversed Baicalin-Induced Autophagy and Abolished Its Protective Role on Mitochondrial Apoptotic Pathway

**FIGURE 5:**
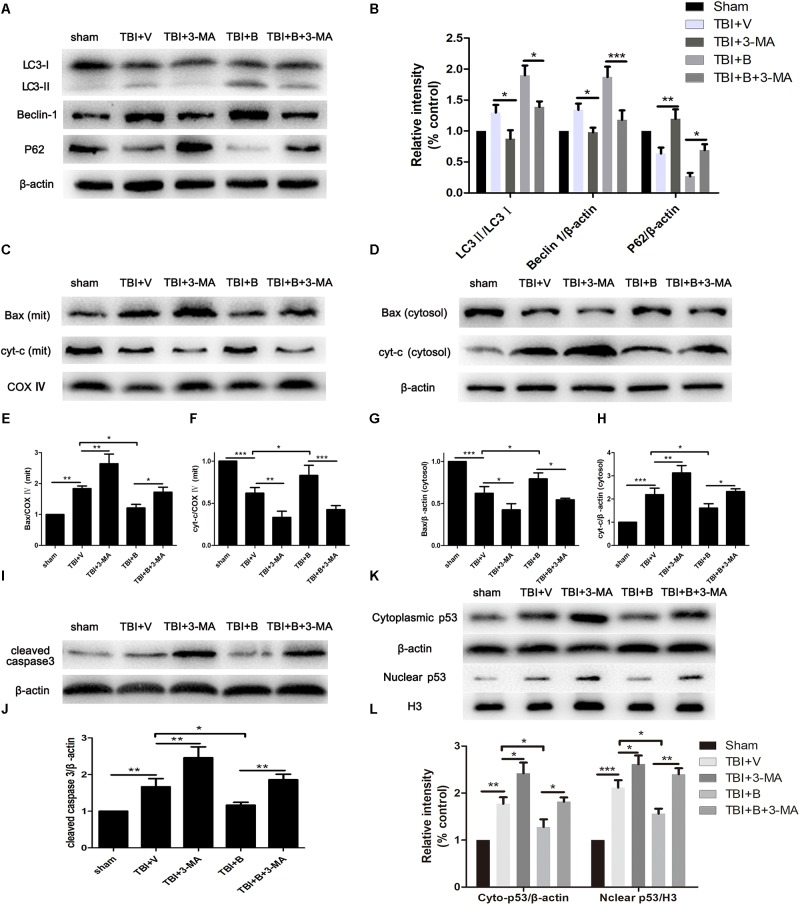
3-MA inhibited autophagy and reversed Baicalin-induced neuroprotection on mitochondrial apoptosis. Mice were divided into five groups: sham, TBI+ V, TBI+ 3-MA, TBI+ B, TBI+ B+ 3-MA. **(A,B)** The western blot band and semiquantitative analysis of LC3, Beclin 1, and p62. **(C,E,F)** The western blot band and semiquantitative analysis of Bax and cyt-c in mitochondrial fraction. **(D,G,H)** The western blot band and semiquantitative analysis of Bax and cyt-c in cytoplasmic fraction. **(I,J)** The western blot band and semiquantitative analysis of cleaved caspase 3. **(K,L)** The western blot band and semiquantitative analysis of cytoplasmic and nuclear p53. Data are represented as mean ± SD (*n* = 6 per group). ^∗^*P* < 0.05, ^∗∗^*P* < 0.01, and ^∗∗∗^*P* < 0.001.

Then we applied 3-methyladenine (3-MA) to investigate the relationship between the Baicalin-mediated autophagy and the apoptosis in TBI. 3-MA is a commonly used autophagy inhibitor that suppressed the formation of autophagosome at the initiation stage by interacting with Vps34. When 3-MA applied, we observed significant decreases of LC3 II and Beclin 1 in TBI+ 3-MA and TB+ B+ 3-MA groups, as compared to TBI+ V (*p* < 0.05 and *p* < 0.05; Figures 5A,B) and TBI+ B (*p* < 0.05 and *p* < 0.001; Figures 5A,B) groups, respectively. However, the protein level of p62 was increased after 3-MA treatment as compared to TBI+ V (*p* < 0.01; Figures 5A,B) and TBI+ B (*p* < 0.05; Figures 5A,B) groups. The results indicated that the autophagy activation caused by TBI or Baicalin was inhibited by 3-MA.

Then we detected the markers of mitochondria apoptosis pathway to further explore the mechanism of neuroprotection of autophagy in TBI. The molecular process of mitochondrial-dependent apoptosis can be simply depicted as the translocation of pro-apoptotic proteins such as Bax from cytoplasm to mitochondrial membrane and the release of cytochrome C from mitochondria to cytoplasm, resulting to cleavage of caspase-3, an executor of apoptosis (Wei et al., 2015).

As shown in Figures 5C,D, the level of Bax was much higher in mitochondria and lower in cytoplasm in TBI mice as compared with sham controls (*p* < 0.01 and *p* < 0.001; Figures 5E,G). Meanwhile, cytochrome C was significantly increased in cytoplasm but decreased in mitochondria after TBI (*p* < 0.001 and *p* < 0.001; Figures 5F,H). The results indicated that TBI insult increased the translocation of Bax from cytoplasm to mitochondria and led to the release of cytochrome C from mitochondria to cytoplasm. However, Baicalin restored the translocation of Bax and cyt-c as compared to TBI+ V group in mitochondrial (*p* < 0.05 and *p* < 0.05; Figures 5E,F) and in cytosolic (*p* < 0.05 and *p* < 0.05; Figures 5G,H) fractions, indicating that Baicalin alleviated TBI-induced mitochondrial apoptosis. More importantly, 3-MA significantly enhanced mitochondrial apoptosis on Bax (*p* < 0.01 and *p* < 0.05; Figures 5E,G) and cytochrome C (*p* < 0.01 and *p* < 0.01; Figures 5F,H) and abolished Baicalin-induced restoration of the translocation of Bax (*p* < 0.05 and *p* < 0.05; Figures 5E,G) and cytochrome C (*p* < 0.001 and *p* < 0.05; Figures 5F,H). Collectively, the results demonstrated that 3-MA aggravated mitochondrial apoptosis after TBI and abolished Baicalin-induced neuroprotection on mitochondrial apoptotic pathway.

Consistent with the results of Bax, cyt-c, the protein level of cleaved caspase-3 was increased after inhibition of autophagy in TBI+ 3-MA and TBI+ B+ 3-MA groups as compared to TBI+V (*p* < 0.01; Figures 5I,J) and TBI+ B (*p* < 0.01; Figures 5I,J) groups, respectively.

**FIGURE 6:**
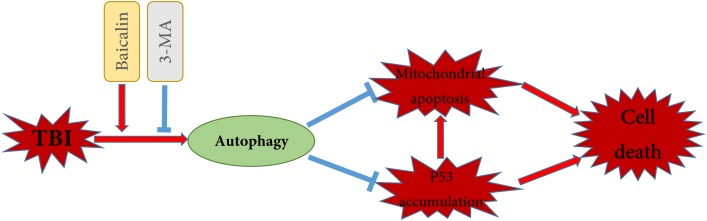
Schematic illustration of the possible neuroprotective mechanisms of Baicalin administration after TBI. As illustrated, Baicalin enhanced TBI-induced autophagy, leading to the reduction of p53 accumulation and the inhibition of mitochondrial apoptosis. In contrast, inhibition of autophagy by 3-MA aggravated p53 accumulation and mitochondrial apoptosis, exacerbating cell death. The red arrows indicate induction, while the blue blocked lines indicate repression.

It has been documented that p53 promotes apoptosis through transcriptional activation of specific target genes or by directly affecting the mitochondrial apoptotic pathway (Green and Kroemer, 2009). Besides, there is a link between activation of the autophagy and p53 degradation in certain cell lines (Kang et al., 2016; Tan et al., 2018). To further explore the possible interaction between autophagy and apoptosis in TBI, we measured the levels of p53 in cytoplasm and nucleus (Figure 5K). The western blot results showed that TBI significantly enhanced the accumulation of p53 both in nuclear and cytoplasmic fractions as compared to sham group (Figure 5L). However, Baicalin decreased p53 accumulation from TBI insult. Furthermore, we found that 3-MA treatment abolished the effect that Baicalin had on p53. The results suggested that p53 accumulation induced by TBI may be degraded by autophagy activated by Baicalin, thus alleviating mitochondrial apoptotic pathway (Figure 6).

## Discussion

TBI is a heterogeneous disease involving complicated pathophysiologic changes. Baicalin is a multi-target drug with a variety of protective effects against brain injury (Zuo et al., 2016; Shi et al., 2017). Recently, our lab has found that Baicalin exerted anti-oxidative neuroprotection against TBI in mice model via Nrf2 pathway (Fang et al., 2018). In this study, we further confirmed the protective role of Baicalin in TBI mice though evaluating neurobehavioral function, brain water content, and BBB permeability. More importantly, we revealed that Baicalin enhanced autophagy after TBI via western blot and immunofluorescence, subsequently inhibited mitochondrial apoptosis. Moreover, 3-MA inhibited Baicalin-induced autophagy and abrogated its neuroprotection on mitochondria apoptosis pathway, which provided a new insight of autophagy as a therapeutic target of Baicalin against TBI.

The prognosis of TBI patients is associated with neuronal cell loss caused by necrosis and apoptosis. Necrosis mostly takes place immediately after TBI due to the mechanical impact. Apoptosis leads to more severe neuronal death as a result of injury-induced secondary injury and takes place from minutes to weeks following injury, which makes it sensitive to therapeutic interventions (Ding et al., 2015). Previous research reported that when apoptosis was inhibited by therapeutic drugs or caspase-3 inhibitor, about a 10% reduction in lesion volume was observed at 24 h and a 30% reduction was observed at 3 weeks after TBI (Clark et al., 2000; Zhang et al., 2018). Our results were consistent with previous studies, the mice treated with Baicalin had a roughly 15% reduction in apoptosis index from the vehicle-treated controls, therefore performing better in motor function evaluation at days 1 and 3.

In the past 10 years, autophagy has been a research hotspot in TBI due to the close relationship between autophagy and cell death. It is now generally believed that autophagy is activated after TBI insult (Diskin et al., 2005; Clark et al., 2008; Luo et al., 2011), but its role in TBI remains controversial. The early study noted that the Beclin 1/TUNEL overlapped cells observed at the lesion site indicated that autophagy could contribute to cell death (Diskin et al., 2005). This view was then supported by several papers demonstrating that inhibition of autophagy by neuroprotective drugs could exert neuroprotection in TBI model (Lai et al., 2008; Wang et al., 2017). However, more recent research suggested that TBI-induced enhancement of autophagy played a protective role in cell survival. Viscomi et al. (2012) demonstrated that decreasing autophagy flux in autophagy deficient Becn1+/- mice resulted in worse functional outcomes than the wild type controls. The opinion that activation of autophagy is neuroprotective and helps reducing neuronal apoptosis in TBI has also been proven in studies using rapamycin (Erlich et al., 2007; Heras-Sandoval et al., 2014), a known autophagy inducer, as well as other drugs (Xu et al., 2014; Ding et al., 2015; Zhang et al., 2016). In the present study, we found that Baicalin administration significantly enhanced autophagy by detecting protein levels of LC3, Beclin 1, and p62, as well as LC3 immunofluorescence. Besides, significantly better motor function recovery and less neuronal apoptosis were observed in Baicalin-treated group, which was consistent with previous studies that support the protective role of autophagy in TBI.

In our opinion, the possible reasons for the dual role of autophagy in TBI are as follows: (1) The degree of autophagy activation is different as there are various environment and stimulus on brain trauma. For example, mild autophagy is beneficial to neuronal survival, as it contributes to the generation of adenosine triphosphate (ATP). In contrast, excessive autophagy results in autophagic cell death or apoptosis (Zhang and Wang, 2018). (2) The drugs used for regulation of autophagy are different. For example, some of treatments used to alleviate autophagy for neuroprotective effects are not specific inhibitors of autophagy, such as ketamine (Wang et al., 2017) and γ-glutamylcysteinyl ethyl ester (GCEE) (Lai et al., 2008). They are given without knowing whether actual autophagy flux is increased or decreased after injection. For instance, the signs of decreased autophagy might be detected because these treatments actually increased autophagy flux and promoted the clearance of accumulated autophagosomes (Lipinski et al., 2015).

The reasons why activated autophagy could reduce apoptosis in TBI are complicated and have not been fully elucidated. It has been documented that increased autophagy flux eliminated TBI-induced damaged mitochondria and accumulation of p53 that were detrimental to cell survival (Lipinski et al., 2015; Tan et al., 2018). There are some other possible mechanisms reported in several papers. Autophagy could sequester the unfolded proteins that initiated endoplasmic reticulum (ER) stress (Fernandez et al., 2015), so activation of autophagy after injury could suppress ER stress and contribute to reducing apoptosis (Lee et al., 2015). In addition, autophagy may also remove TBI-induced toxic ubiquitin-positive protein aggregates, and provide nutriments or energy for the initiation of restorative processes after injury (Loane et al., 2009). Furthermore, Luo provided a perspective, stating that the BIM, a BH3-only protein, could interact with both Bcl2 and Beclin 1. In the stress conditions, BIM was phosphorylated by MAPK8/JNK and dissociated from Beclin 1, which ameliorated autophagy inhibition and activated anti-apoptosis Bcl2 protein (Luo and Rubinsztein, 2013). Nevertheless, the precise mechanism that autophagy has on apoptosis in TBI remains unclear.

In our study, we found that Baicalin activated autophagy and reduced cell apoptosis. What’s more, 3-MA inhibited Baicalin-induced autophagy and abrogated its neuroprotection on mitochondria apoptosis pathway, indicating autophagy activation reduced neuronal apoptosis possibly though eliminating damaged mitochondria after TBI. The results were in accordance with previous studies (Zhang et al., 2013; Cavallucci et al., 2014; Ding et al., 2015), in which they noted that clearance of damaged mitochondria by mitophagy alleviated mitochondrial-dependent apoptosis, therefore promoting cell survival.

To sum up, our studies demonstrated that Baicalin improved mice motor performance, ameliorated brain edema, reduced disruption of BBB, and alleviated mitochondrial apoptosis in the mice TBI model, at least partly via activation of autophagy. These results showed that Baicalin is a promising drug for the treatment of TBI.

## Author Contributions

JF designed the study, performed the biochemical analysis, and wrote the manuscript. YiZ performed the TBI model, prepared the drug solutions, and performed the histological examination. MF, WN, and XL contributed to the western blotting. YuZ, MZ, and XW performed the TUNEL staining and the animal studies. BC revised and checked the manuscript for spelling and grammatical errors. HW contributed to the design and analysis of the study and wrote the manuscript. All authors approved the final version of the manuscript.

## Conflict of Interest Statement

The authors declare that the research was conducted in the absence of any commercial or financial relationships that could be construed as a potential conflict of interest.
